# Association between infant post-natal prophylaxis regimens and pretreatment HIV drug resistance in infected infants

**DOI:** 10.3389/fepid.2026.1887821

**Published:** 2026-08-26

**Authors:** Seth C. Inzaule, Leonard Kingwara, Alani Sulaimon Akanmu, Raph L. Hamers, Michael R. Jordan, Amalia Girón-Callejas, Rukia S. Madada, Lilly Nyagah, Vera Onwonga, Martina Penazzato, Silvia Bertagnolio, Ajay Rangaraj, Kamene Kimenye, Tobias F. Rinke de Wit, Andrew Mula

**Affiliations:** 1 Amsterdam Institute for Global Health and Development, and Department of Global Health, Amsterdam UMC, University of Amsterdam, Amsterdam, Netherlands; 2 Kenya National Public Health Institute, Ministry of Health, Nairobi, Kenya; 3 Department of Haematology and Blood Transfusion, Lagos University Teaching Hospital, and College of Medicine of the University of Lagos, Lagos, Nigeria; 4 Faculty of Medicine, Oxford University Clinical Research Unit Indonesia, Universitas Indonesia, Jakarta, Indonesia; 5 Centre for Tropical Medicine and Global Health, Nuffield Dept of Medicine, University of Oxford, Oxford, United Kingdom; 6 Levey Center for Integrated Management of Antimicrobial Resistance, Tufts University School of Medicine, Boston, MA, United States; 7 Division of Geographic Medicine and Infectious Diseases, Tufts Medical Center, Boston, MA, United States; 8 Independent Researcher, Guatemala, Guatemala; 9 National AIDS and STI Control Programme, Ministry of Health, Nairobi, Kenya; 10 World Health Organization, Geneva, Switzerland

**Keywords:** HIV drug resistance, HIV vertical transmission, infants living with HIV, post-natal infant prophylaxis, sub-Saharan Africa, pre-treatment HIV drug resistance

## Abstract

**Background:**

Antiretroviral drugs for preventing mother-to-child transmission (PMTCT) can lead to NRTI resistance in infants who contract HIV despite prophylaxis. We aimed to assess how different post-natal prophylaxis (PNP) strategies; nevirapine (NVP) alone vs zidovudine (ZDV) alone vs dual NVP + ZDV influenced NRTI resistance prevalence.

**Methods:**

We analyzed data from nationally representative HIV drug resistance surveys conducted in Kenya where the standard practice was the use of dual NVP + ZDV and Nigeria where the standard practice was the use of NVP PNP. HIV drug resistance was determined using population-based sequencing, and mutations interpreted using the Stanford HIVdb algorithm. Analyses were weighted for multistage sampling and genotyping success rates.

**Results:**

Among 557 HIV-infected infants aged <18 months with data on resistance and type of PNP, 29.3% received dual NVP + ZDV PNP, 24.6% received NVP alone, 2.3% received ZDV alone, and 43.8% received no PNP. The prevalence of NRTI resistance was 19.4% (95%CI 15.2–24.3) in Nigeria and 9.6% (95%CI 6.6–13.8) in Kenya (*p* < 0.001). Resistance to abacavir, lamivudine, zidovudine and tenofovir were 2- to 4.5-fold higher in Nigerian HIV infected infants exposed to NVP PNP compared to Kenyan infants exposed to NVP + ZDV. The proportion of infants with NNRTI resistance was 49% (95%CI 43.5–54.5) and 43.8% (95%CI 38.0–49.7), in Nigeria and Kenya respectively (*p* = 0.204).

**Conclusions:**

These findings suggest an association between type of PNP and the development of NRTI resistance in HIV-infected infants. Dual-drug prophylaxis was associated with a low prevalence of NRTI resistance compared to single-drug PNP. Our findings highlight the need for further research on combination prophylactic strategies in minimizing the emergence of drug resistance in this population.

## Introduction

The scale-up of interventions to prevent mother-to-child HIV transmission (PMTCT) including providing combination antiretroviral therapy (ART) for pregnant HIV-positive women, and nevirapine and/or zidovudine prophylaxis for HIV-exposed infants has led to a 61% decline in new paediatric HIV infections globally by 2024 compared with 2010 levels of 310,000 infections (1).

Although the expansion of PMTCT programmes has substantially reduced the incidence of paediatric HIV infection, it has inadvertently contributed to high levels of pretreatment drug resistance (PDR) among the small proportion of children who acquire HIV despite maternal ART and/or infant antiretroviral (ARV) prophylaxis (2–4). Drug resistance observed in infants with HIV infection may arise from exposure to sub-therapeutic concentrations of ARV drugs through breast milk, exposure to infant post-natal prophylaxis (PNP), or transmission of resistant viral strains from the mother (5–7). PDR in infants has been associated with poor treatment outcomes, including virological failure, progression to AIDS, and increased mortality (8–10).

To improve treatment outcomes among HIV-infected infants, the World Health Organization (WHO) has recommended since 2013 that children younger than three years be transitioned from non-nucleoside reverse transcriptase inhibitor (NNRTI)-based regimens to protease inhibitor (PI)-based regimens, and more recently to dolutegravir (DTG)-containing regimens, due to the high prevalence of pretreatment NNRTI resistance in this population (11). Recent studies, however, have documented an increasing prevalence of pretreatment resistance to nucleos(t)ide reverse transcriptase inhibitors (NRTIs). This trend appears to be associated with the widespread rollout of lifelong ART and PMTCT strategies for mothers (2, 12). Although studies in adults suggest that DTG-based regimens remain highly effective even in the presence of background NRTI resistance when combined with tenofovir and lamivudine, it remains uncertain whether similar efficacy will be observed in infants, for whom the recommended NRTI backbone is abacavir (ABC)-based therapy. Furthermore, emerging evidence suggests that background NRTI resistance may increase the risk of treatment failure and the emergence of DTG resistance (13). Therefore, preventing NRTI resistance in infants remains critical in the DTG era to ensure sustained long-term effectiveness of the limited ART options available for this population.

Since 2016, WHO has recommended nevirapine (NVP) prophylaxis for six weeks for HIV-exposed infants whose mothers are receiving ART (14). For infants at high risk of HIV acquisition, an enhanced post-natal prophylaxis (ePNP) strategy consisting of six weeks of NVP plus zidovudine (ZDV), followed by an additional six weeks of NVP or NVP plus ZDV in breastfeeding infants, was recommended (14). More recently in 2025, WHO recommends the use of three-drug regimen comprising ABC/3TC + DTG as the preferred ePNP regimen for high-risk infants during the first six weeks, followed by extended use of a single-drug regimen with NVP as the preferred option or DTG or 3TC as alternatives beyond the initial six weeks of triple-drug PNP and until maternal viral suppression is achieved or breastfeeding has ceased (15). At the time of implementation of this study, the standard practice in Nigeria was to use NVP single prophylaxis for low-risk infants and NVP + ZDV for high-risk infants (16). On the other hand, the standard in Kenya was to use NVP + ZDV dual PNP for all infants regardless of risk followed by six weeks of NVP PNP (17). Since then, the guidelines have changed with Kenyan guidelines recommending the use of triple ARV prophylaxis (14 weeks of ABC+3TC + DTG followed by NVP until six weeks after cessation of breastfeeding), for high-risk infants and using NVP + ZDV for low-risk infants for six weeks followed by NVP until six weeks after complete cessation of breastfeeding.

The objective of this analysis was to compare the prevalence of NRTI resistance in infants exposed to NVP + ZDV in Kenya, (2018) and in infants exposed to NVP PNP in Nigeria (2016), where ePNP is restricted to high-risk infants only.

## Methods

We analyzed data from nationally representative surveys conducted in accordance with WHO guidance for HIV drug resistance surveillance, specifically targeting infants aged <18 months who were newly diagnosed with HIV through early infant diagnosis (EID) testing programmes (18). We included all EID laboratories in each country and aimed for a target sample size of 500 as per the WHO recommendations (18). Where fewer than 500 eligible specimens were available, we included all eligible specimens. Samples were allocated to EID laboratories proportionally to the number of eligible HIV-positive specimens available during the 12-month survey period and were selected within each laboratory using systematic sampling.

The HIV-1 *pol* gene was genotyped using Sanger-based method at a WHO-designated laboratory, and sequences were quality-assured following WHO recommendations. Genotyping success rate was 72% for Nigeria and 70% for Kenya. HIV drug resistance was predicted using the Stanford HIVdb algorithm Version 9.0. Sequences classified as having low-, intermediate- or high-level resistance were categorized as resistant. Prevalence estimates of HIV drug resistance were assessed using the ’svy’ utilities in Stata (Stata 12; StataCorp, College Station, Texas, USA). Analyses were weighted to adjust for multistage sampling and genotypic failure rate. We used logistic regression to explore the association between PNP regimen and PDR. Approval for non-research determination of routine surveillance data was obtained from the National Health Research Ethics Committee, Nigeria and the University of Nairobi-Kenyatta National Hospital Ethical Review Committee, Kenya as a routine surveillance study.

## Results

Of 959 infants diagnosed with HIV, 703 had data on resistance and were included in the study. Of these 385 (54.8%) were from Kenya and 318 (45.2%) from Nigeria. Data on type of PNP were available for 557 (79%); 314/318 (98.7%) of infants from Kenya and 243/385 (63%) from Nigeria. Of the 314 infants with data on PNP from Kenya, 51.9% (163) had received NVP + ZDV while 4.8% (15) had been exposed to NVP while 43% (136) had not received any PNP (Table 1). On the other hand, 50.2% (122/243) of infants from Nigeria had received NVP alone, 5.3% (13/125) to ZDV, while 58.4% (142/243) had not been received any PNP. The median age was 5 months (IQR 2–9.6) and 51% (284) were female.

**Table 1:** Prevalence of NRTI resistance in treatment-naive children less than 18 months of age exposed to dual ZDV + NVP PNP in Kenya compared to those exposed to single NVP-based PNP in Nigeria.

Infant prophylaxis	95%CI					
	NNRTI resistant	NRTI resistance	ABC resistance	3TC resistance	ZDV resistance	TDF resistance
Prevalence %						
All (*n* = 703)	44.0 (40.0–47.3)	15.6 (13.0–18.3)	13.1 (10.6–15.5)	10.5 (8.3–12.8)	6.5 (4.7–8.4)	5.0 (3.4–6.6)
NVP + ZDV (*n* = 163)	49.7 (41.9–57.5)	9.2 (4.7–13.7)	6.7 (2.9–10.6)	5.5 (2.0–9.1)	4.3 (1.1–7.4)	4.3 (1.1–7.4)
NVP (*n* = 137)	50.4 (41.9–58.9)	24.8 (17.6–32.1)	21.2 (14.3–28.1)	19.0 (12.4–25.5)	10.9 (5.7–16.2)	8.0 (3.4–12.6)
ZDV (*n* = 13)	38.4 (12.2–64.5)	46.2 (14.8–77.5)	46.2 (15.0–77.5)	30.8 (17.4–59.8)	15.4 (0.0–38.1)	15.4 (0.0–38.1)
None (*n* = 244)	61.5 (30.9–92.1)	9.0 (5.4–12.6)	7.0 (3.7–10.2)	5.3 (2.5–8.2)	3.7 (1.3–6.1)	2.5 (0.5–4.4)
Missing	50.4 (41.9–58.8)	24.8 (17.5–32.1)	21.2 (14.2–28.1)	19.0 (12.3–25.6)	10.9 (5.6–16.2)	8.0 (3.4–12.6)
Kenya						
All (*n* = 318)	43.4 (37.9–48.9)	8.8 (5.7–11.9)	6.3 (3.6–9.0)	4.4 (2.1–6.7)	4.1 (1.9–6.3)	3.5 (1.4–5.5)
NVP + ZDV (*n* = 163)	49.7 (41.9–57.5)	9.2 (4.7–13.7)	6.7 (2.9–10.6)	5.5 (2.0–9.8)	4.3 (1.1–7.4)	4.3 (1.1–7.4)
NVP (*n* = 15)	46.7 (18.1–75.3)	6.7 (0.0–21.0)	6.7 (0.0–21.0)	_	_	_
None (*n* = 136)	36.0 (27.9–44.2)	8.8 (4.0–13.7)	5.9 (1.9–9.9)	3.7 (0.5–6.9)	4.4 (0.9–7.9)	2.9 (0.1–5.8)
Missing	_	25.0 (0.0–100)	_	_	_	_
Nigeria						
All (*n* = 385)	43.9 (38.9–48.9)	21.3 (17.2–25.4)	18.7 (14.8–22.6)	15.6 (11.9–19.2)	8.6 (5.8–11.4)	6.2 (3.8–8.7)
NVP (*n* = 122)	50.8 (41.8–59.8)	27.0 (19.0–35.0)	23.0 (15.4–30.5)	21.3 (13.9–28.7)	12.3 (6.4–18.2)	9.0 (3.9–14.2)
ZDV (*n* = 13)	38.4 (12.2–64.5)	46.2 (14.8–77.5)	46.2 (15.0–77.5)	30.8 (17.4–59.8)	15.4 (0.0–38.1)	15.4 (0.0–38.1)
None (*n* = 142)	36.1 (26.9–45.3)	9.3 (3.7–14.8)	8.3 (3.0–13.6)	7.4 (2.4–12.4)	2.8 (0,0–5.9)	1.9 (0.0–4.4)
Missing (*n* = 108)	42.3 (34.0–50.5)	23.2 (16.2–30.3)	20.4 (13.7–27.1)	15.5 (9.5–21.5)	9.2 (4.4–14.0)	6.3 (2.3–10.4)

3TC: lamivudine; ABC: abacavir; CI: confidence interval; NNRTI: non-nucleoside reverse transcriptase inhibitor; NRTI: nucleoside reverse transcriptase inhibitor; NVP: nevirapine; OR: odds ratio; PNP: post-natal prophylaxis; TDF: tenofovir disoproxil fumarate; ZDV: zidovudine.

The overall prevalence of pretreatment NRTI resistance was 15.6% (95%CI 13.0–18.3), being 9.2% (95%CI 4.7–13.7) for infants on dual NVP + ZDV PNP, 24.8% (95%CI 17.6–32.1) for NVP only, 46.2% (95%CI 14.8–77.5) for ZDV PNP only and 9.0% (95%CI 5.4–12.6) for those not on any PNP. The proportion of NRTI resistance in Kenya was 8.8% (95%CI 5.7–11.9) and 21.3% (95%CI 17.2–25.4) (*p* *<* *0.001*) in Nigeria. Compared with infants receiving dual NVP + ZDV PNP, the risk of NRTI resistance was 3.3 times higher among those who received NVP PNP only (OR 3.26, 95%CI 1.68–6.30), 8.5 times higher among those who received ZDV PNP only (OR 8.46, 95%CI 2.51–28.55). No significant difference was observed among infants who did not receive any prophylaxis (OR 0.98, 95% CI 0.49–1.95) (Table 2).

**Table 2:** Exploratory analysis comparing the prevalence of NRTI resistance in treatment-naive children less than 18 months of age exposed to dual ZDV + NVP PNP compared to those exposed to either NVP or ZDV single PNP in national representative surveys in Kenya and Nigeria.

Infant prophylaxis	95%CI					
	NNRTI resistant	NRTI resistance	ABC resistance	3TC resistance	ZDV resistance	TDF resistance
**OR**						
NVP + ZDV (*n* = 163)	1.0	1.0	1.0	1.0	1.0	1.0
NVP (*n* = 137)	1.03 (0.65–1.62)	3.26 (1.68–6.30)	3.71 (1.77–7.77)	4.01 (1.80–8.91)	2.74 (1.08–6.95)	1.95 (0.73–5.18)
ZDV (*n* = 13)	1.62 (0.51–5.19)	8.46 (2.51–28.55)	11.8 (3.34–41.52)	7.60 (1.95–29.64)	4.05 (0.75–22.0)	4.05 (0.75–22.0)
None (*n* = 244)	0.57 (0.38–0.86)	0.98 (0.49–1.95)	1.03 (0.47–2.28)	0.96 (0.40–2.31)	0.85 (0.31–2.35)	0.56 (0.18–1.71)

3TC: lamivudine; ABC: abacavir; CI: confidence interval; NNRTI: non-nucleoside reverse transcriptase inhibitor; NRTI: nucleoside reverse transcriptase inhibitor; NVP: nevirapine; OR: odds ratio; PNP: post-natal prophylaxis; TDF: tenofovir disoproxil fumarate; ZDV: zidovudine.

Overall, pretreatment NRTI resistance was driven mainly by resistance to abacavir (ABC) and lamivudine/emtricitabine (XTC), regardless of PNP type (Table 1). The prevalence of ABC and XTC resistance in Nigeria was 18.7 (14.8–22.6) and 15.6 (11.9–19.2) respectively compared to 6.3% (95%CI 3.6–9.0) and 4.4% (95%CI 2.1–6.7) in Kenya. On the other hand, the prevalence of tenofovir (TDF) and ZDV resistance was 6.2% (95%CI 3.8–8.7) and 8.6% (95% CI 5.8–11.4) in Nigeria compared with 3.5% (95%CI 1.4–5.5) and 4.1% (95%CI 1.9–6.3) in Kenya. On the other hand, the prevalence of ABC, XTC, ZDV and TDF resistance was 46.2% (95%CI 15.0–77.5), 30.8% (95% CI 17.4–59.8), 15.4% (95%CI 0.0–38.1) and 15.4% (95%CI 0.0–38.1) respectively for those on ZDV PNP in Nigeria. Among infants who were not on any PNP, the prevalence of NRTI resistance was 9.0% (95% CI 5.4–12.6), being 8.8% (95%CI 4.0–13.7) in Kenya and 9.3% (95%CI 3.7–14.8) in Nigeria.

On the other hand, the prevalence of NNRTI resistance was 44.0% (95%CI 40.0–47.3) being 49.7% (95%CI 41.9–57.5) for those on dual NVP + ZDV PNP, 50.4% (95%CI 41.9–58.9) for those on NVP PNP alone, 38.4% (95%CI 12.2–64.5) for those on ZDV PNP alone. The prevalence of NNRTI resistance among infants who did not receive any PNP was 36.1% (95%CI 30.0–42.1) being 36.0 (95%CI 27.9–44.2) in Kenya and 36.1% (95%CI 26.9–45.3) in Nigeria.

In a sensitivity analysis to determine the effect of missing PNP information, we observed similar patterns of NRTI and NNRTI resistance being, 24.8% (95%CI 17.5–32.1) (being 6.7%, 95%CI 0.0–21.0 in Kenya and 27.0%, 95%CI 19.1–35.0, in Nigeria) and 50.4% (95%CI 41.9–58.8) (being 46.7%, 95%CI 18.1–75.3 in Kenya and 50.8%, 95%CI 41.8–59.8, Nigeria) respectively.

There was no statistically significant difference in risk of developing NNRTI resistance by type of PNP (Table 2). Compared to infants who were on a dual NVP + ZDV PNP, the risk of NNRTI resistance was 1.03 (95%CI, 0.65–1.62) for those on mono NVP PNP, and 1.62 (95%CI, 0.51–5.19) among those on mono ZDV PNP. Infants who had not received any PNP had a 43% lesser risk of developing NNRTI resistance compared to those on dual NVP + ZDV PNP (OR 0.57, 95%CI 0.38–0.86) (Table 1).

**Figure 1:**
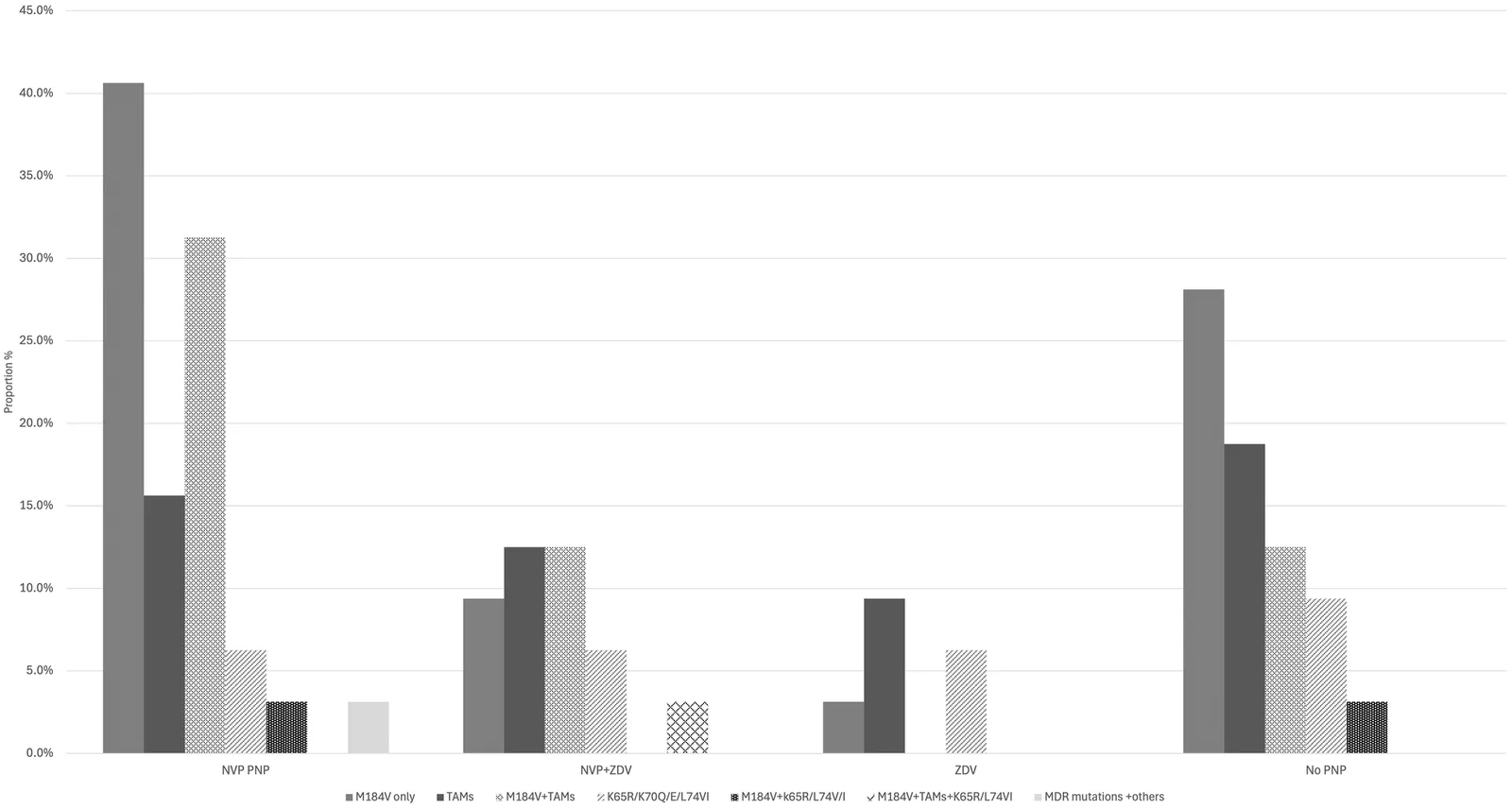
Drug resistance mutations patterns among newly diagnosed infants <18 months exposed to ZDV + NVP dual PNP compared to infants exposed to either ZDV or NVP single PNP in Kenya and Nigeria MDR: multi-drug resistance; NVP; nevirapine, PNP: post-natal prophylaxis; TAMs: thymidine analogue mutations; ZDV: zidovudine; TAMs included M41L, D67G/N, K70R, L210W, T215A/I/Y/F and K219Q/E/R; MDR included Q151M., F116Y, V75I.

The pattern of NRTI resistance also differed by type of PNP and mainly comprised thymidine analogue mutations (TAMs) with or without M184 V (64.3%, 9/14) for infants exposed to ZDV + NVP PNP, and only 21.4% (3/14) was due to M184IV only. On the other hand, 41% (13/32) of NRTI resistance among infants exposed to NVP PNP was attributed to M184IV alone, whereas 50% (16/32) was still due to the presence of TAMs with or without M184 V (Figure 1). Similarly, TAMS with or without M184 V were observed in 50% (3/6) of the infants exposed to ZDV-only PNP (Figure 1).

## Discussion

In this analysis of nationally representative HIV drug resistance survey data from Kenya and Nigeria, we observed higher levels of NRTI resistance among HIV-infected infants who received single-drug post-natal prophylaxis (NVP or ZDV) compared with those who received dual NVP + ZDV prophylaxis. Resistance was predominantly driven by mutations affecting ABC and the cytosine analogues 3TC and FTC. These findings suggest an association between infant prophylaxis regimen and the prevalence of NRTI resistance among infants diagnosed with HIV among the small proportion of infants who acquire HIV infection despite PMTCT interventions.

The relatively high levels of NRTI pretreatment resistance are concerning, as there is uncertainty in the efficacy of regimens containing a compromised ABC containing NRTI backbone when co-administered with DTG or PI's. Recent studies have shown that DTG and PIs remain efficacious even when used in combination with tenofovir disoproxil fumarate (TDF) or ZDV in the setting of predicted TDF or ZDV resistance (19, 20). The efficacy of DTG or PIs in the presence of a compromised TDF containing backbone has been attributed to residual activity of the NRTIs, the high genetic barrier of DTG and PIs and an antagonistic effect of the M184 V mutation, which causes hypersusceptibility of HIV to TDF (19, 20). On the contrary, it increases resistance to ABC especially when it occurs together with TAMs as is the case in majority of the infants in this study (21, 22). Emerging evidence also suggests that pre-existing NRTI resistance (13) and the use of abacavir (23) may increase the risk of development of resistance to dolutegravir. These concerns highlight the importance of minimizing the development of NRTI resistance during PMTCT and infant prophylaxis strategies in order to preserve the long-term efficacy of limited pediatric antiretroviral treatment options. More studies are needed to further assess the impact of ABC resistance on treatment outcomes when used in combination with PI/r and integrase inhibitors.

Our findings further suggest that dual NVP + ZDV PNP may reduce the emergence of NRTI resistance compared with single-drug prophylaxis among the small proportion of infants who acquire HIV despite PNP. We hypothesize that the high prevalence of NRTI resistance observed in this study may be explained by infant exposure to sub-therapeutic concentrations of maternal ART through breast milk. The frequent detection of TAMs, including among infants receiving NVP PNP alone, suggest post-infection selection of resistance during ongoing exposure to low maternal ART concentrations ingested during breastfeeding. These observations are consistent with prior PMTCT studies showing that ART-naïve infants exposed to maternal ART may initially be infected with wild-type virus but subsequently develop resistance following sustained exposure to low ARV drug concentrations during breastfeeding (7, 24, 25). In this setting, extended combination prophylaxis with NVP + ZDV may mitigate resistance selection by increasing the genetic barrier to resistance and limiting replication of partially suppressed virus.

Although it is likely that some NRTI resistance may have been transmitted at the time of infection, we observed a lower prevalence of NRTI resistance among infants receiving dual NVP + ZDV PNP. This finding supports the hypothesis that combination prophylaxis reduces resistance selection in infants who become infected despite PMTCT interventions.

Although our study showed a high risk of NRTI resistance with ZDV-based PNP, these findings should be interpreted with caution due to the small sample size. Overall, the limited sample size is insufficient to make any conclusive inferences on the association between ZDV single PNP and risk of NRTI resistance in this population. However, it is worth noting that the recent WHO recommendations no longer recommend the use of ZDV-based PNP due to its toxicity.

Taken together, these findings suggests that the use of dual or triple antiretroviral prophylaxis in HIV-exposed infants may not only help to reduce transmission but also to preserve future treatment options for those who become infected despite PNP. While safety considerations remain important, the high mortality associated with untreated infant HIV infection and the need for lifelong therapy underscore the importance of prophylactic strategies that minimize the emergence of antiretroviral resistance.

This study has several limitations. First, the cross-sectional design precludes differentiation between resistance transmitted during infection and resistance selected after infection due to infant prophylaxis or exposure to sub-therapeutic maternal ART through breast milk. Second, due to the retrospective nature of the study, important confounders such as maternal ART regimen, viral load, drug resistance, infant infection and duration of breastfeeding were not collected though they have been postulated to be associated with PDR in infants (24) Thirdly, our study design comparing data from two countries is amenable to ecological bias, and the observed difference in the prevalence of NRTI resistance may be due to other uncontrolled confounders unique to each setting such as maternal non-adherence and subsequent transmission of NRTI resistant virus. Caution is therefore warranted in the interpretation of these findings. Fourth, our analyses on association of type of PNP with NRTI resistance was only exploratory and we could not account for differences in PMTCT programme histories between Kenya and Nigeria as well as differences in maternal ART regimens, and surveillance periods which may impact the findings. Fifth, these findings may evolve with the widespread use of dolutegravir-based regimens in mothers, as studies indicate that dolutegravir can also be transferred through breast milk at low concentrations (25), and its influence on neonatal resistance requires further evaluation. Sixth, information on PNP exposure was missing for a substantial proportion of infants, which may introduce bias. However, we observed the same pattern of NRTI and NNRTI prevalence between Nigeria and Kenya among infants with missing PNP information. Finally, the cross-sectional design does not allow direct assessment of causality between prophylaxis regimens and the emergence of resistance.

Despite these limitations, this study from nationally representative surveillance data suggests the potential role of combination infant prophylaxis regimens in preventing the emergence of NRTI resistance and the subsequent impact on pediatric HIV treatment. As PMTCT programs continue to evolve and vertical transmission rates decline, optimizing prophylaxis strategies to minimize the emergence of drug resistance will remain essential.

In conclusion, our exploratory analysis show that combination PNP may be associated with a lower risk of NRTI resistance among infants who acquire HIV infection despite PMTCT interventions. Further research is needed to evaluate whether combination PNP offers greater benefits than single-drug regimens in preventing NRTI resistance during the era of integrase inhibitor–based therapy.

## Data Availability

The datasets presented in this study can be found in online repositories. The names of the repository/repositories and accession number(s) can be found below: https://www.ncbi.nlm.nih.gov/genbank/, MH801446-MH801869.
